# The promise of energy-efficient battery-powered urban aircraft

**DOI:** 10.1073/pnas.2111164118

**Published:** 2021-11-02

**Authors:** Shashank Sripad, Venkatasubramanian Viswanathan

**Affiliations:** ^a^Department of Mechanical Engineering, Carnegie Mellon University, Pittsburgh, PA 15213

**Keywords:** energy efficiency, transport electrification, urban air mobility

## Abstract

Improvements in rechargeable batteries are enabling several electric urban air mobility (UAM) aircraft designs with up to 300 mi of range with payload equivalents of up to seven passengers. Novel UAM aircraft consume between 130 Wh/passenger-mi and ∼ 1,200 Wh/passenger-mi depending on the design and utilization, compared to an expected consumption of over 220 Wh/passenger-mi and 1,000 Wh/passenger-mi for terrestrial electric vehicles and combustion engine vehicles, respectively. We also find that several UAM aircraft designs are approaching technological viability with current Li-ion batteries, based on the specific power and energy, while rechargeability and lifetime performance remain uncertain. These aspects highlight the technological readiness of a new segment of transportation.

Aircraft designed to travel up to 300 mi are currently used for various applications, including mobility of passengers and cargo as well as security and emergency services via helicopters or small planes. Recently, urban air mobility (UAM) has emerged as a platform that could transform transportation in urban areas and displace services of terrestrial vehicles. UAM concepts hinge on the development of electric vertical takeoff and landing (EVTOL) aircraft. These aircraft operate using “vertiports” (similar to helipads) with no runway, making them particularly suitable for urban environments. EVTOL aircraft also present a twofold to sixfold faster means of point-to-point mobility compared to terrestrial alternatives (1). Due to these attributes of UAM, large investments amounting to several billion US dollars have been mobilized in 2021 (2).

Aircraft electrification enables distributed (electric) propulsion since electric motor efficiency and power density are scale invariant, unlike combustion engines (3). A large number of small electric motors could be used instead of conventional combustion-based propulsion architectures with a few (less than four) relatively large propulsion units (3, 4). Distributed propulsion reduces drag significantly (3, 4), while electric motors are about twofold to threefold more efficient than combustion engines, resulting in considerably higher overall efficiency for electric aircraft (4).

Over the last few years, several novel UAM aircraft designs have emerged, enabled by the improvements in specific energy and power associated with Li-ion batteries (4). The UAM aircraft design space comprises a highly diverse set of specifications for cruising distance, maximum takeoff mass (MTOM), payload capacity, and rate of energy consumption. There are three broad categories of EVTOL aircraft: 1) multirotor, similar to helicopters but with multiple rotors distributed over an aircraft, generally without a fixed wing; 2) lift plus cruise, where one set of rotors are used for takeoff and landing (vertical flight) and another set are used for cruising, generally with a fixed wing; and 3) vectored thrust, generally fixed-wing aircraft where the thrust-providing system of the aircraft is used in both vertical and forward flight by maneuvering the direction of thrust. Vectored thrust can be further categorized into 3a) tilt rotor, where rotors used in vertical flight tilt via rotating shafts to be used in forward flight; 3b) tilt wing, where the tilting action is performed by wings onto which the rotors are attached; and 3c) tilt duct, similar to tilt rotor but with the thrust generated by propellers that are housed within cylindrical ducts, sometimes called ducted fans.

The power requirement in vertical flight is strongly influenced by a design parameter called “disk loading” (kilograms per square meter) which is the ratio of MTOM to total rotor disk area (5). Horizontal flight power requirements are influenced strongly by the “lift-to-drag ratio” (L/D) (5), as shown in *Materials and Methods*. Multirotors and aircraft with a larger total rotor disk area, resulting in a lower disk loading, require lower power for takeoff and landing (5). On the other hand, designs with a low total rotor disk area require high vertical flight power. Multirotors with large rotors cause an increase in drag leading to high power requirements during cruise. Aircraft with fixed wings that provide lift during cruise have higher energy efficiency in horizontal flight.

Notwithstanding the differences in power requirements due to design parameters like disk loading or L/D ratio, across all EVTOL designs, the energy consumption per unit mile traveled for takeoff, landing, and hovering segments with a considerable vertical component is much higher than the cruise segment. Therefore, to a first approximation, the total energy consumption per unit mile for a trip is directly proportional to the fraction of time spent in vertical flight. For fixed takeoff and landing segments, once an aircraft reaches the specified flying altitude, as the cruise distance increases, the overall energy consumption per unit mile for the trip generally decreases (with an optimum at a certain cruising speed; *SI Appendix*). Previous studies (1, 6) have used fixed values for parameters such as disk loading, resulting in estimates failing to describe several new aircraft designs (7, 8).

To compare the energy efficiency of terrestrial vehicles like electric vehicles (EVs) and EVTOL aircraft, certain differences between the two modes need to be accounted for. EVTOLs cover point-to-point distance without meanders, whereas EVs travel on roads with circuitous paths resulting in a longer distance covered between the same points. Previous studies have suggested global circuity factors between 1.12 and 2.10, while the US average route circuity is about 1.20 (1). Another important factor is the number of occupants or the amount of payload carried by the vehicles. In the United States, the average occupancy for light vehicles including motorcycles, cars, and light trucks has been 1.67 for over 10 y (9). Hence, the appropriate metric to compare energy efficiency, in this context, is the energy consumption per unit distance per unit payload carried. Watt-hour per passenger-mile after accounting for circuity is used for terrestrial vehicles.

We choose five EVTOL aircraft representative of the diverse EVTOL aircraft design space: 1) Kitty Hawk Corporation (KH) Heaviside (tilt rotor), 2) Joby Aviation (Joby) 2021 (yet to be named aircraft) (tilt rotor), 3) Lilium GmbH (Lilium) Jet (tilt duct), 4) Beta Technologies (Beta) Alia-250 (lift plus cruise), and 5) Archer Aviation (Archer) Maker (lift plus cruise/tilt rotor); these are designed to carry one, five, seven, six, and two passengers (including the pilot, if used) while traveling 100, 150, 172 (150 nautical mi), 288 (250 nautical mi), and 60 mi, respectively. A previously developed EVTOL power consumption model, described in *Materials and Methods* and *SI Appendix*, is used to analyze these aircraft (10).

## Results and Discussion

**Fig. 1:**
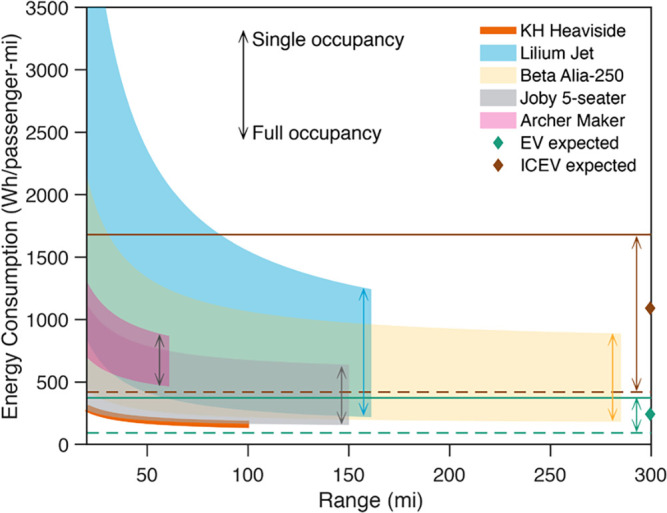
Energy efficiency of different EVTOL aircraft and terrestrial vehicles. The diamond markers represent the expected EV and ICEV at an occupancy of 1.67 (9). The energy consumption for all EVTOL aircraft is estimated at a cruising speed of 150 mi/h which is up to sixfold faster than equivalent terrestrial vehicles (1). Energy consumption for single-passenger KH Heaviside is occupancy invariant. As the length of the cruise segment increases, the energy consumption decreases. Fully occupied EVTOLs are equivalent to or more energy efficient than fully occupied ICEVs for flying ranges of more than 70 mi or lower depending on the aircraft, while the energy consumption is similar to or lower than an expected EV (223 Wh/passenger-mi) after 100 mi.

In Fig. 1, across all aircraft considered, as the length cruise segment increases with longer flying range, the efficiency improves drastically. For a single passenger, the energy consumption of larger aircraft is generally higher, Lilium Jet ¿ Beta Alia-250 ¿ Archer Maker ≈ Joby 5-seater ¿ KH Heaviside. Lilium Jet uses ducted fans resulting in high energy consumption for vertical flight due to the high disk loading, but, as the cruise length increases, the energy consumption drops rapidly compared to other aircraft, due to its highly efficient cruising segment.

Fig. 1 describes the energy consumption comparison of the five aircraft with a terrestrial EV and internal combustion engine vehicle (ICEV). The EV and ICEV are assumed to have a fixed duty cycle over the travel distances analyzed and are examined at single, maximum, and expected occupancy. The details of the estimates for EV and ICEV are provided in *SI Appendix*. At median occupancy, all five aircraft are more efficient at designed range than the expected ICEV (1,000 Wh/passenger-mi). At full occupancy and designed range, all aircraft are more efficient or equivalent to a fully occupied ICEV (420 Wh/passenger-mi). Beyond 20 mi, the KH Heaviside is always more efficient than an ICEV irrespective of the ICEV occupancy considered.

On comparing the efficiency of EVs with the five aircraft, we find that the single-passenger KH Heaviside is more efficient than an EV with one occupant at ranges greater than 20 mi and more efficient than the expected EV after cruising 35 mi. A fully occupied Joby five-seater, Beta Alia-250, and Lilium Jet show an energy consumption of about 156, 161, and 218 Wh/passenger-mi, respectively, at their designed flying range, all lower than the expected EV at 223 Wh/passenger-mi. This represents a significant energy efficiency milestone for EVTOL aircraft, highlighting the enormous efficiency gains that can be achieved via fixed-wing cruising.

One of the crucial enabling factors for modern EVTOL aircraft, as noted previously, is the battery pack (4). There has been tremendous progress in performance and cost of Li-ion and related battery chemistries over the last decade. However, earlier studies on EVTOL aircraft (1, 8) include fixed cell-level specific energy assumptions for batteries and/or no consideration of specific power, thereby missing the interplay between aircraft design parameters and battery requirements.

Given the advanced thermal management systems that exist in modern aircraft, some EVTOL manufacturers have proposed approaches to designing battery packing and management systems that are integrated with other onboard systems, thereby improving the pack-level specific energy (7, 11). The MTOM of an electric aircraft can be broadly divided into three parts: 1) payload, 2) battery weight, and 3) empty weight that accounts for the weight of the aircraft structures, airframe, propulsion systems, and other onboard systems.

**Fig. 2:**
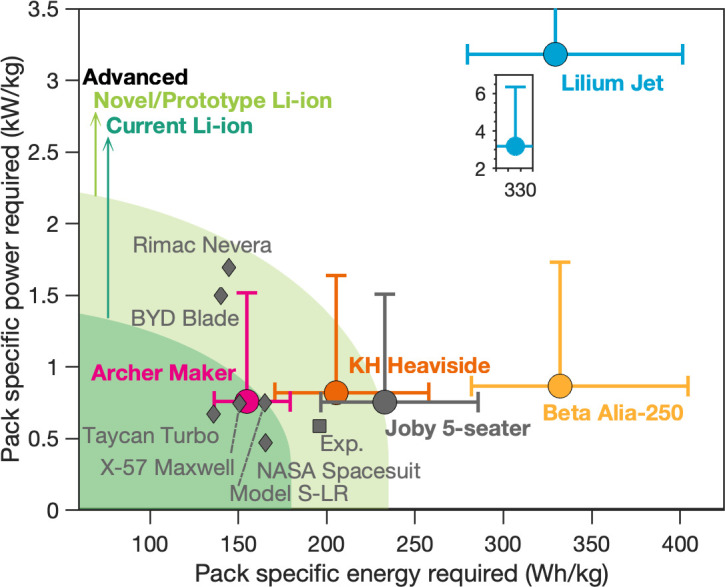
Pack specific energy and specific power (discharge) requirements for the aircraft analyzed at an EWF of 0.5, where the abscissa error bars indicate estimates at an EWF of 0.45 and 0.55. Cruising speed for maximum range with 30-min reserves is assumed for battery sizing. The ordinate error bars show the landing power requirement where half the battery pack has failed. Battery packs that have been developed, to date, are shown and labeled as gray diamonds. “Current Li-ion” represents batteries manufactured at large scale; “Novel/prototype Li-ion” indicates chemistries and designs developed recently or for high-performance applications; “Advanced” indicates nascent pack designs that are not yet commercially available. The gray square labeled “Exp.” shows the only experimental EVTOL battery reported in literature, reported by Yang et al. (6). *Inset* shows the zoomed in pack specific power and pack specific energy for the Lilium Jet.

In Fig. 2, we explore the battery pack specific energy and specific (discharge) power requirements, as defined by the range and takeoff/landing power demands for the five EVTOL aircraft. A comparison with several currently available battery pack designs in EVs, experimental (X) planes, and space applications is shown (see *SI Appendix* for dataset). In Fig. 2, we show three categories for battery pack technology based on technical readiness and commercial availability as well as the only reported battery designed for EVTOL applications in academic literature (6).

The battery pack specific energy requirements in Fig. 2 are estimated using an empty weight fraction (EWF) of 0.5 [lower than current aviation standards (10)] to facilitate the possible use of battery packing weight for structures and other shared functions (7, 11). Estimates are shown using uncertainty bounds for EWF of 0.45 and 0.55 reflected as the abscissa error bars. A lower EWF provides more weight allocation for the battery, thereby reducing the required battery performance metrics.

Aircraft like the Lilium Jet that have a high disk loading (*SI Appendix*) require higher power for takeoff, landing, and hover compared to other designs. Coupled with a high MTOM, the specific power requirements for the Lilium Jet are much higher than for other aircraft, as seen in Fig. 2. Longer flying range requires larger battery packs, resulting in higher specific energy requirements for aircraft like the Beta Alia-250. On the other hand, low-range aircraft like Archer Maker require much lower specific energy, and such designs are feasible with current Li-ion batteries. The importance of EWF can be observed by examining the increase in specific energy required for each aircraft to accommodate a higher EWF of 0.55. The uncertainty limits for power account for the possibility of partial failure of the battery pack in a scenario where only 50% of the battery pack supplies the total required power to land. The strong influence of EWF and battery pack failure on specific energy and power requirements shows that regulations could play an important role in determining the technical viability of EVTOL aircraft.

Fig. 2 emphasizes the importance of specific power being a more critical performance metric for EVTOLs which determines whether an EVTOL can safely take off and land. On the other hand, to a first approximation, specific energy determines the operating range of the EVTOL. It should also be noted that Fig. 2 does not make provisions for degradation in performance metrics, and the values could be considered the minimum required performance at the end of life, especially for specific power, given that the ability to land is safety critical. Other aspects related to battery behavior during high power requirements during landing are not reflected in Fig. 2, and relevant discussions can be found elsewhere (10). In the overall analysis, in Fig. 2, we find that several EVTOL designs can achieve the promising energy efficiency shown in Fig. 1 via suitable improvements to current Li-ion batteries, while charging and performance over lifetime require further investigation. This highlights the technological readiness of EVTOL aircraft, from the battery technology standpoint.

In this brief report, we have discussed two main details: 1) the energy efficiency of EVTOL compared to terrestrial vehicles and 2) battery requirements compared to the current battery technological landscape. We noted the technological readiness in terms of battery requirements. The promise of EVTOL aircraft achieving higher energy efficiencies than equivalent terrestrial alternatives at faster travel times signals enormous implications for the emission intensity and sustainability of urban transportation.

## Materials and Methods

The vertical flight power for open rotor aircraft is given by

$${\text{P}}_{\text{vertical,open}}=[\frac{\text{f}\;\text{W}}{\text{FoM}}\sqrt{\frac{\text{f}\;\text{W/A}}{2\;\rho }}+\frac{\text{W}\;{\text{V}}_{\text{climb,v}}}{2}]/\eta_{\text{vertical}}$$

and, for ducted fan aircraft, is given by

$${\text{P}}_{\text{vertical,ducted}}=[\frac{\text{f}\;\text{W}}{2\;\text{FoM}}\sqrt{\frac{\text{f}\;\text{W/A}}{\rho }}+\frac{\text{W}\;{\text{V}}_{\text{climb,v}}}{2}]/\eta_{\text{vertical}}$$

The figure of merit (FoM) is the ratio between ideal and actual rotor power (12); f is a correction factor for interference from the fuselage, and is set to the typical value 1.03 (12). The disk area, A, determines the disk loading. Density of air (*ρ*) is calculated at flight altitude. $V_{\mathrm{climb},v}$ is the climb rate, and, when held at zero, corresponds to “hover” conditions. Aircraft weight (W) is the product of MTOM and acceleration due to gravity. $\eta_{\text{vertical}}$ is the combined efficiency of the motors and electric powertrain during vertical flight conditions. Fixed-wing segment power (12) (${\text{P}}_{\text{fixed}-\text{wing}}$) is given by

$${\text{P}}_{\text{fixed}-\text{wing}}=[\text{W}\;{\text{V}}_{\text{v}}+\frac{\text{W}\;\text{V}}{\left[\text{L/D}\right]}]/(\eta_{\text{fixed}-\text{wing}}).$$

$\eta_{\text{fixed}-\text{wing}}$ includes the efficiency of the propellers as well. The vertical velocity component (${\text{V}}_{\text{v}}$) is zero for cruise. The L/D and the forward velocity (V) are segment specific. The operating conditions are estimated using a minimum power condition for climb and descent. Extended methods can be found in *SI Appendix*.

## Data Availability

Aircraft parameters and battery data are available in *SI Appendix* and hosted at GitHub, https://github.com/BattModels/evtol (13).
